# Yeast epigenetics: the inheritance of histone modification states

**DOI:** 10.1042/BSR20182006

**Published:** 2019-05-07

**Authors:** Callum J. O’Kane, Edel M. Hyland

**Affiliations:** School of Biological Sciences, Queen’s University Belfast, Belfast, U.K.

**Keywords:** chromatin, epigenetics, Histone Modifications, Saccharomyces cerevisiae, Schizosaccharomyces pombe

## Abstract

*Saccharomyces cerevisiae* (budding yeast) and *Schizosaccharomyces pombe* (fission yeast) are two of the most recognised and well-studied model systems for epigenetic regulation and the inheritance of chromatin states. Their silent loci serve as a proxy for heterochromatic chromatin in higher eukaryotes, and as such both species have provided a wealth of information on the mechanisms behind the establishment and maintenance of epigenetic states, not only in yeast, but in higher eukaryotes. This review focuses specifically on the role of histone modifications in governing telomeric silencing in *S. cerevisiae* and centromeric silencing in *S. pombe* as examples of genetic loci that exemplify epigenetic inheritance. We discuss the recent advancements that for the first time provide a mechanistic understanding of how heterochromatin, dictated by histone modifications specifically, is preserved during S-phase. We also discuss the current state of our understanding of yeast nucleosome dynamics during DNA replication, an essential component in delineating the contribution of histone modifications to epigenetic inheritance.

## Yeast as a model system for studying epigenetics

The term epigenetics has invoked much controversy since it was coined by Conrad Waddington in 1942 [1] and despite our best efforts, a working definition of epigenetics varies widely [2]. For the purposes of this review, we define an epigenetic phenomenon as one that meets the following two criteria: (i) it impacts the structure of chromatin, thereby altering the phenotype of a cell, independent of changes to the underlying DNA sequence. (ii) The altered chromatin state, or a memory of the state, is heritable at least through S-phase.

In the last two decades, studies on yeast have shaped much of our current understanding of epigenetic mechanisms, and they are likely to continue to refine that understanding particularly regarding inheritance of epigenetic states. First and foremost, the characteristics that afforded yeast its pivotal role throughout the genomic era [3] apply to its utility in epigenetic research. These include its short generation time, small compact genome, well-characterised genetics and reproductive cycle, the availability of an extensive number of sophisticated genetic technologies, and more recently, systems biology information (reviewed in [4]). In addition, however, yeast also offers one of the simplest eukaryotic epigenomes, which is a commodity given the innate complexity of epigenetics. The epigenetic systems of yeast, in particular *Saccharomyces cerevisiae*, represent a reductionist version of higher eukaryotic systems. DNA CpG methylation is absent from both budding and fission yeasts, and *S. cerevisiae* also lacks the RNA interference (RNAi) machinery and repressive histone H3K9 methylation, which are found in fission yeast and many other eukaryotes. Comparative analyses of gene body DNA methylation in eukaryotes suggest that these yeast lineages lost this ancient epigenetic pathway early in their evolution, and thus rely primarily on histone modification patterns to demarcate epigenetic states [5]. Although this may appear to limit the viability of yeast as a model system, the absence of these processes simplifies the examination of the remaining epigenetic marks. Indeed, over the last decade it has become apparent that cross-talk exists between the DNA methylation and histone modification pathways in animal cells [6], which can complicate the study of either process. The lack of DNA methylation in yeast simplifies the examination of the relationship between histone modifications and chromatin-based epigenetic states.

Furthermore, budding and fission yeasts are arguably the best available models for studying the inheritance of histone modification-dependent chromatin states, through cell division. *S. cerevisiae* and *Schizosaccharomyces pombe* contain only two and three functionally redundant copies of the genes encoding each core histone protein respectively, whereas mammalian and insect cells typically contain >50 copies [7,8]. As such, yeast strains containing a single copy of each histone gene can be easily engineered, simplifying both the mutagenic analysis of histone proteins, and histone-tagging experiments. This ease of histone genetics, in combination with the short generation times, maximise the practicality of yeast as a model system to experimentally assess the inheritance of epigenetic marks.

To date, epigenetic phenomena in yeast are restricted to the repressive transcriptionally silent loci that bear many of the hallmarks of heterochromatin in higher eukaryotes. The importance of these silent states in yeast is highlighted by the fact that in the absence of such epigenetic repression, both *S. cerevisiae* and *S. pombe* are unable to mate and complete the entirety of their life cycles. Although the epigenetic processes operating in budding yeast and fission yeast share some similarities, they represent distinct phenomena, and can be used as a paradigm for the epigenetics of numerous other cellular systems. A comparison of epigenetic mechanisms between *S. cerevisiae* and *S. pombe* is found in Table 1. In *S. cerevisiae*, these epigenetic phenomena include (i) Telomere Silencing, the variegated expression pattern of subtelomeric genes; (ii) Mating Type Silencing, the constitutive epigenetic repression of the silent mating loci; and (iii) rDNA Silencing, the partial repression of RNA pol II-transcribed genes within rDNA arrays. Similarly, epigenetic silencing in *S. pombe* occurs at its telomeres and at the mating-type locus. Additionally, the pericentromeric regions of *S. pombe* chromosomes are subject to transcriptional repression. This review focuses on the role of histone modifications in yeast transcriptional silencing and details our current understanding of how these marks contribute to the inheritance of epigenetic chromatin states.

**Table 1:** Comparison of the key features of telomeric silencing in *S. cerevisiae* and centromeric silencing in *S. pombe*

	*S. cerevisiae* telomeric silencing	*S. pombe* centromeric silencing
Sequence-specific establishment site	TG1–3 repeats and telomere ends	Primarily the ∼4 kb *cenH* sequence
Recruitment factor	Rap1p and yKup	Interactivity between RNAi and CLRC
Histone PTM delineating silent state	Deacetylated H4K16	Methylated H3K9
DNA-sequence requirement for spreading?	No	No
Protein bound to modified histones	SIR complex	HP1p
Positive feedback loop	Co-operativity between Sir2p, Sir3p, and H4K16ac	Co-operativity between RNAi loop and CLRC self-recruitment
Continuous silencing	No	Yes
Antagonising enzymes	Dot1p (methyltransferase), Sas2p (acetyltransferase), Rpd3Lp (deacetylase)	Lsd1p (demethylase), Epe1p (demethylase)
Empirical evidence of sequence-independent inheritance?	No	Yes

Abbreviations: H3K9, Histone H3 lysine 9; H4K16Ac, histone H4 lysine 16 acetylation; SIR, silent information regulator.

## Establishment of telomeric silencing in *S. cerevisiae*

**Figure 1:**
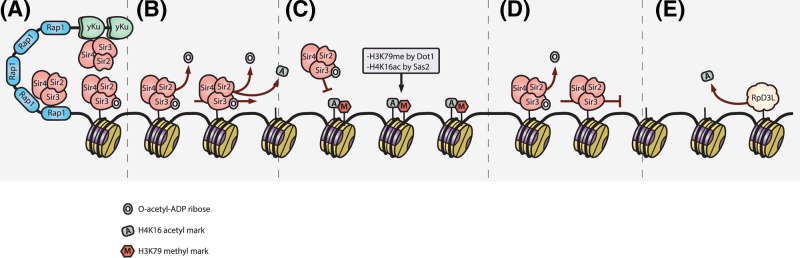
Telomere silencing in *S. cerevisiae* (**A**) Heterochromatin formation is nucleated by the recruitment of the SIR complex to the telomere ends by the DNA binding proteins Rap1p and yKu70/80p. (**B**) The SIR complex spreads along the telomere by a deacetylation-enforced positive feedback loop. (**C**) Specific barrier elements such as the activity of Dot1p and Sas2p antagonise SIR spreading. (**D**) Sir protein occupancy at telomeres is discontinuous. (**E**) The RpD3L deacetylase complex also antagonises SIR spreading, and SIR spreading may be opposed by a competing positive feedback loop. Abbreviation: SIR, silent information regulator. RESOLUTION is not optimal. Will send a higher resolution file.

The sustained silencing of certain genes within telomeric chromatin in *S. cerevisiae* is one of the most well-characterised examples of how epigenetic information is stored and inherited, and we will use it to describe the distinctive properties of epigenetic loci. Although telomeric silencing was initially believed to be non-discriminatory, more recent high-resolution studies indicate that silencing at telomeres is more discrete and occurs in a non-continuous fashion along the length of the chromosome ends [9]. In *S. cerevisiae*, silent loci are devoid of the majority of histone modifications suggesting that their contribution to the heterochromatin-like state is minimal. However, at telomeres it is the active removal of specific histone marks that demarcate silent from ‘non-silent’ chromatin, emphasising their key role in regulating the establishment and stability of this epigenetic locus (summarised in Figure 1).

As with all epigenetic loci, the heterochromatic state of telomeres in *S. cerevisiae* is due to the action of epigenetic marks that function independently of the telomeric DNA sequence. That said however, the establishment phase does rely on sequence-specific DNA binding proteins namely Rap1p and the yKu70p/yKu80p heterodimer, which bind the telomeric TG_1–3_ repeats, and the chromosome end respectively [10,11]. These telomere-binding proteins recruit the silent information regulator (SIR) complex consisting of Sir2p, Sir3p, and Sir4p in equal stoichiometry [12]. The transcriptionally silent state results from the spreading of this SIR complex along a portion of the telomere driven by co-operative interactions between Sir2p, Sir3p, and the histone proteins, irrespective of the underlying DNA sequence (Figure 1).

Initially, Sir4p is recruited to telomeres via a direct interaction with Rap1p [13] at a so-called nucleation site or silencer, and serves as a scaffold for the correct assembly of the remainder of the SIR complex. Once assembled, the co-operative binding and spreading of the SIR complex along telomeres is primarily regulated by two specific histone modifications, namely histone H4 lysine 16 acetylation (H4K16ac) and histone H3 lysine 79 methylation (H3K79me). This is primarily because Sir3p selectively binds hypoacetylated H4K16 nucleosomes and this binding is inhibited by the presence of H3K79me [14,15]. Indeed specific domains of Sir3p have been mapped that mediate these nucleosomal contacts. The Sir3p C-terminal domain recognises the acetylation status H4K16 acetylation and binds Rap1p and Sir4p accordingly. The Sir3p bromo-adjacent-homology (BAH) domain contacts the nucleosome surface encompassing H3K79, and the strength of this interaction is critical in establishing telomeric silencing. Therefore, the modification of H4K16 and H3K79, or lack thereof, chemically defines the chromosomal locations that facilitate Sir3p interaction, promoting the specificity of SIR complex binding (reviewed in [16]).

Sir2p directly influences histone modification states as it possesses NAD-dependent histone deacetylase activity (reviewed in [17]). Sir2p is preferentially recruited to nucleosomes marked with histone H4K16ac [18] targeting them for deacetylation and thereby generating high affinity binding sites for Sir3p. This deacetylation reaction forms O-acetyl-ADP-ribose (OAADPr) as a by-product, which increases the affinity of the heterotrimeric SIR complex at these nucleosomal binding sites [19]. Therefore, the Sir2p-mediated deacetylation of nearby histone proteins generates a positive feedback mechanism that facilitates the transition from nucleation to polymerisation of the SIR complex along telomeres [11,20–22]. Not surprisingly, a deletion of *SIR2* or a catalytic mutant of Sir2p prevents SIR complex spreading and abrogates telomeric silencing in *S. cerevisiae* [9,20,21]. Similarly, the transcriptional regulation of *SIR2* can also impact heterochromatin spreading, for example the repression of *SIR2* by heat shock results in the euchromatinisation of subtelomeric regions over multiple generations [23].

## Bistability of telomeric chromatin states in *S. cerevisiae*

The insertion of reporter genes into subtelomeric locations has permitted the study of the stability and heritability of telomeric silent chromatin. In 1990, Gottschling et al. [24] showed that once established, telomeric silent chromatin exerts a heritable, variegating effect on gene expression, known as the telomeric position effect (TPE). *S. cerevisiae* cells with a telomere-linked *ADE2* reporter gene produced colonies that were predominantly either white (*ADE2* expressing) with red (*ADE2* repressed) sectors, or red with white sectors. This indicated that genetically identical cells give rise to two distinct heritable *ADE2* phenotypes that are dependent on the chromatin state of *S. cerevisiae* telomeres, underscoring the epigenetic nature of telomeric silencing. The variegated expression pattern of *ADE2* within individual colonies however indicates a level of instability or switching between these chromatin states during mitotic cell division. Given that the ‘ON’ state is also heritable it suggests that telomeric silencing is *not* routinely re-established based on the DNA sequence after every cell division. Indeed, single cell analysis in yeast has shown that the establishment of telomeric silencing occurs stochastically over several generations [25]. Therefore, this suggests that an opposing system to repress the activity of the SIR complex exists that maintains the ‘ON’ state. Much work in the last 15 years has cumulated in a model dictated by the interplay between the histone modifications H4K16ac and H3K79me that underlie this bistability of telomeric gene expression.

In order to control the spread of heterochromatin outside telomeres, mechanisms exist that interfere with the positive feedback mechanism employed by the SIR complex. The recruitment of the histone acetyltransferase Sas2p to telomere proximal sites, and the subsequent acetylation of H4K16 disrupts the activity of the SIR complex by antagonising Sir3p binding [26]. Furthermore, the activity of Sas2p favours the recruitment of histone methyltransferase Dot1p, which preferentially binds to acetylated histone H4K16 of and methylates H3K79 [27,28]. This core methylation serves to positively reinforce the inhibition of silencing by weakening the interaction of Sir3p to nucleosomes. Sas2p-mediated acetylation of H4K16 is also thought to enhance the incorporation of the histone H2A variant Htz1/H2AZ [29], which may act as an additional barrier to SIR complex spreading [30]. Therefore, it is not surprising that (i) the deletion of *SAS2* leads to the spreading of the silencing complex approximately five-fold further than in a wild-type cell [26,31] and (ii) the overexpression of *SAS2* leads to the loss of Sir3p binding to telomeres and a loss of silencing [27]. Furthermore, in *dot1Δ* strains telomeric silencing is compromised, as the SIR complex relocalises to other genomic locations, limiting its availability at telomeres [32] (Figure 1).

Intriguingly, the histone deacetylase complex Rpd3L is also thought to delineate the barriers of silent chromatin. It was found that the simultaneous disruption of both the catalytic component of the Rpd3L complex as well as *SAS2* results in heterochromatin spreading to the point of lethality [33]. A possible explanation for this is that in normal cells the non-discriminant deacetylase activity of Rpd3p removes substrates required for the spread of the SIR complex. Therefore, without acetyl substrates, Sir2p is unable to produce OAADPr, thus losing an important driver for SIR complex propagation outside true heterochromatic regions. Alternatively, the Rpd3p-mediated removal of marks on histones H2B and H4 may enforce a chromatin configuration that is impermeable to the SIR complex [34]. Taken together, it is understood that the fine-tuned balance of Sas2p, Rpd3p, and Dot1p, and their associated histone modifications, likely determine the boundaries of silent chromatin, alongside factors such as the activity of specific barrier elements, [35] the availability of Sir proteins [36], certain chromatin remodelling activity [37], and the spatial arrangement of nucleosomes [38].

It is also noted that other euchromatic-associated histone modifications, such as histone H3K36me [39] and H3K4me [40], have been shown to influence silencing boundaries in *S. cerevisiae*. Similar to H3K79 methylation they act indirectly to inhibit SIR complex binding to euchromatin, and therefore are thought to play a predominantly redundant role in telomeric silencing [41]. However, further analysis is required to support these initial findings.

The direct role of histone modifications in telomeric silencing is supported through the analysis of *S. cerevisiae* histone mutations at the sites of modification. Accordingly, the mutations H4K16A and H3K79A lead to a loss of telomeric silencing (*lts*) phenotype, as determined by the expression of genes at subtelomeric locations [42,43]. Interestingly, mutations that mimic the constitutively non-modified state of both these sites, H4K16R and H3K79R exhibit different phenotypes. H4K16R gives rise to an *lts* phenotype due to the diluting of SIR complex to other genomic locations [31] and H3K79R shows an increased silencing phenotype *(its*) [44] potentially due to an enhanced binding of SIR complex to telomeric locations specifically. These contrasting results suggest that non-methylated H3K79 is *not* sufficient by itself to recruit the SIR complex to non-silenced euchromatic locations, whereas constitutively deacetylated H4K16 can.

## The inheritance of telomeric chromatin states in *S. cerevisiae*

How are these distinct transcriptional states at telomeric loci inherited, and what is the molecular basis of switching? (See review [45]). True epigenetic inheritance would rely on the faithful transmission of the chromatin state irrespective of the DNA sequence. If this information is stored primarily by histone modifications, for silent telomeric loci at least it would be the inheritance of modification-free nucleosomes that would perpetuate the ‘OFF’ state in the daughter cells. These nucleosomes could then be self-templating post-replication by recruiting the SIR complex, thus blocking the action of anti-silencers to remain unmodified and/or deacetylating any newly incorporated H4K16ac modified histones. Experiments that support this potential mechanism of heterochromatin inheritance are currently lacking in *S. cerevisiae*, however recent reports in *S. pombe* (detailed below) lend confidence to its feasibility. Inconsistent with this model however, is the observation that removal of the DNA sequences required in the establishment phase of silencing abrogated silencing at the HMR locus in a single cell cycle [46,47]. This indicates that silencing, at HMR locus at least, is re-established with each round of cell division and that histone modification-free nucleosomes cannot by themselves maintain silent chromatin states. However, as mentioned above, the propagation of the ‘ON’ state challenges this assertion, as re-establishment is somehow prevented in these ‘ON’ cells.

The heritability of the transcriptionally ‘ON’ state at *S. cerevisiae* telomeres suggests that the opposing system, which represses the activity of the SIR complex, is stably maintained through cell division. Using chromatin immunoprecipitation (ChIP) it was revealed that the expression state of telomeric chromatin is regulated by the frequency of Dot1p-induced H3K79 methylation, which is prominent in telomeres in the ‘ON’ state [28]. Furthermore, H3K79me seems to be propagated by a transcription-linked positive feedback loop, implying that the epigenetic expression profile of telomeric genes is determined by switching between two competing feedback loops, one dictated by H3K79 methylation and the other by H4K16 deacetylation. The apparent lack of a known H3K79 specific demethylase in *S. cerevisiae* suggests that this histone mark is permanent and therefore inheritance of the ‘ON’ state is dictated by the faithful transmission of H3K79me through mitotic division, an event that is yet to be analysed in detail in yeast. In human cells however, it was suggested that the re-establishment of H3K79 methylation patterns post-replication is kinetically slow [48], unlike other ‘true’ epigenetic marks such as H3K9me that is re-established concomitantly with the deposition of new histones during S-phase [49] suggesting that it is not a faithful transmitter of gene expression states in human cells at least. Further work is needed in yeast to understand the heritability of the ‘ON’ state at a mechanistic level and its proposed dependency on H3K79me.

Similarly, the factors/conditions that induce the switch between the ‘ON’ and ‘OFF’ expression states remain unclear, but it is undeniable that the level of inheritance of the histone marks H4K16ac and H3K79me through S-phase must play a considerable role in the maintenance and inheritance of bistable chromatin states [28]. Theoretically, the suboptimal transmission of H3K79me, and possibly H4K16ac modified nucleosomes might hinder the re-establishment of the ‘ON’ state in daughter cells, promoting a switch to the repressed state, although this remains to be investigated.

Alternatively, the physical location of silent telomeres may influence the heritability of chromatin states [50]. Telomeres are anchored to the perinuclear membrane at foci that contain a high abundance of silencing factors, and it has been proposed that the documented release of telomeres from the nuclear periphery during DNA replication [51], would provide an opportunity to cells to switch between epigenetic states. However, other studies suggest that this localisation is not essential for silencing [52] and moreover, physically tethering chromosomes to the nuclear periphery does not influence TPE substantially, illustrating that by itself, nuclear localisation is not sufficient to promote the silenced state [53]. Therefore, the contribution of telomere localisation to bistability needs to be addressed in light of these studies. Furthermore, given the evidence that the nuclear localisation of individual yeast telomeres can rely on distinct pathways [54], delineating a single mechanism that underlies a potential role in telomeric bistability, might be unrealistic.

## *In silico* modelling of yeast epigenetic inheritance

Given the obvious complexity of bistable epigenetic states in yeast, recent efforts have been focused on generating simplified *in silico* models in order to identify the fundamental features required for this process [55,56]. One such model, based on epigenetic silencing mechanisms in *S. pombe*, showed that robust bistability required (i) co-operativity between multiple modified nucleosomes through the positive feedback loops that propagate histone modifications, and (ii) modified nucleosomes capable of catalysing the modification of non-adjacent nucleosomes [56], highlighting the central role of histone modifications to this phenomenon. In the absence of these features, the model predicted that an epigenetic state would be both unstable and excessively insensitive, as minor changes in histone modifications would stall the system in an intermediate state resulting in the loss of the epigenetic expression profile. More recently, a model of SIR-silencing in *S. cerevisiae* was developed, which corroborated the findings of the aforementioned model, and emphasised the importance of co-operativity in the controlled spreading of the SIR complex [55]. This model also predicted that heritable bistability could be achieved even when the co-operativity between SIR-bound nucleosomes was limited to adjacent nucleosomes, albeit under the assumption that Sir2p can deacetylate distant nucleosomes. Although neither model incorporated the role of histone H3K79 methylation, they clearly show that inheritance of epigenetic states is highly sensitive to the deacetylation of histone H4K16 by the SIR complex.

A more sophisticated model was put forward in 2014 that begins to address the dynamics of H4K16ac and H3K79me modifications and, more specifically, how the two opposing feedback loops operate to accurately dictate expression states of heterochromatin [57]. This result from this model indicates that telomeres are not in fact maintained in distinct ‘ON’ and ‘OFF’ states, but that at threshold concentrations of SIR complex, the ‘OFF’ state merges with the ‘ON’ state suggesting that the cell has the capability of dialling up or down telomeric silencing presumably dictated by its specific needs over time. The authors suggest that this prediction, which is supported by experimental data, could be accounted for given the dynamic nature of chromatin boundaries. Clearly, further investigation is required, before we can elucidate the precise mechanism of inheritance of telomeric states, but combining *in silico* model predictions with yeast genetic approaches, might prove the most successful method to achieve this.

Nonetheless the cumulative findings of practical and theoretical studies qualify telomere silencing as a true epigenetic locus in yeast regulated primarily by histone modification patterns. Indeed, SIR-mediated gene repression exemplifies many of the features that, at a theoretical level, characterise epigenetic loci, including positive feedback looping, barrier elements, and co-operativity. However, in spite of this there still remains mechanistic gaps in our knowledge of how heterochromatin persist through mitosis in *S. cerevisiae*. Thankfully two papers in 2015 focusing on *S. pombe* centromeric silencing have shed light on the answers to these questions [58,59]. These papers were the first to demonstrate how the inheritance of histone modifications alone through S-phase is sufficient to re-establish heterochromatin in newly formed daughter cells, proving that in *S. pombe* at least, epigenetic inheritance can be achieved through the modification of histone proteins.

## Centromeric silencing in *S. pombe*: the inheritance of histone H3K9 methylation

**Figure 2:**
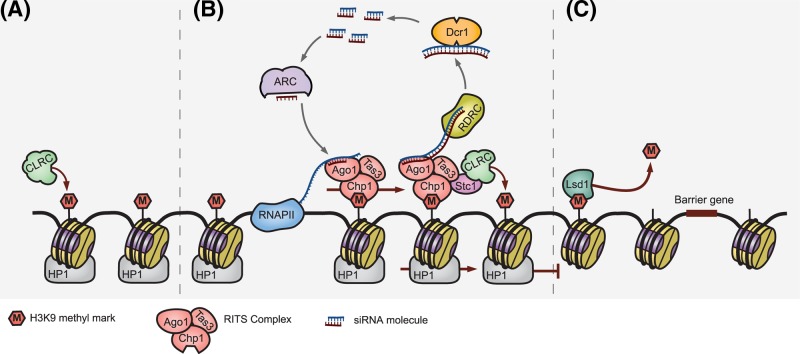
Centromere silencing in *S. pombe* (**A**) Heterochromatin formation is initiated by the deposition of a H3K9me mark by the CLRC complex. The factors leading to the recruitment of CLRC remain unclear. (**B**) The H3K9me mark is propagated by a co-operative interaction between the RNAi feedback loop and CLRC (see text), Swi6p/HP1 binds methylated histones, leading to the silent state. (**C**) Barrier elements such as the activity of Lsd1p and sequence-encoded heterochromatin insulators define the limits of the positive feedback mechanism and therefore, silent chromatin. Abbreviation: CLRC, Clr4p methyltransferase complex. RESOLUTION not optimal - needs to be upgraded.

The mechanisms behind the formation and maintenance of heterochromatin in *S. pombe* are considerably different from those of *S. cerevisiae*. Unlike *S. cerevisiae*, the *S. pombe* genome contains regions of centromeric heterochromatin. This silent chromatin is marked by methylated H3K9 nucleosomes, which are bound by the protein Swi6p/HP1p, maintaining a repressive chromatin state. Additionally, the regulation of centromeric heterochromatin in *S. pombe* requires the activity of the RNAi pathway, which drive the establishment and spread of epigenetic silencing (summarised in Figure 2).

Centromeric heterochromatin in *S. pombe* is initiated by the deposition of a H3K9me mark by the methyltransferase Clr4p, a component of the CLRC complex [60]. Clr4p is also capable of binding H3K9me, meaning that like the SIR complex, CLRC can autonomously propagate its associated histone modification [61]. However, unlike SIR silencing, robust epigenetic repression in *S. pombe* depends on the association of the RNAi-induced transcriptional silencing (RITS) complex. RITS recognises and binds centromeres via an interaction with H3K9me, and in the presence of short-interfering RNA (siRNA) molecules facilitates the spreading of the heterochromatic state. These siRNA molecules are generated through a self-enforcing loop, wherein DNA in heterochromatic regions is transcribed at low levels by RNA Polymerase II (RNAPII). The nascent transcripts serve as a template for RNAi machinery including the RNA-dependent RNA polymerase complex (RDRC), and the Dcr1p enzyme, generating siRNAs. The subsequent binding of the Argonaute siRNA Chaperone (ARC) complex to these siRNA molecules, and converts them into a single-stranded form, ready to be loaded on to the RITS complex. The loading of RITS with siRNA facilitates an interaction with the nascent RNAPII transcripts from which the siRNAs were ultimately derived, thus completing one cycle of the loop (Figure 2). The activated RITS complex also interacts with RDRC, promoting dsRNA production, and with CLRC, enforcing the spread of the H3K9me mark. Although it seems counterintuitive that heterochromatin formation, which antagonises transcription, relies on the activity of RNAPII, it has recently been demonstrated that the extent of H3K9 methylation can influence the heterochromatic state [62] Domains associated with H3K79me2 were shown to be transcriptionally permissive, whereas H3K79me3 delineated a silent state, which was required for the inheritance of silencing. This illustrates that the establishment kinetics of H3K9 methylation levels may define the function and heritability of heterochromatin in *S. pombe.*

Despite the differences between SIR silencing and CLRC silencing, the two processes are fundamentally similar, both are initiated by DNA sequence-specific recruitment, and propagated by ‘read-write’ mechanisms, ultimately facilitating the association of heterochromatin modeling proteins. The ‘read-write’ functionality of these pathways suggests that after their establishment, the silencing machinery is capable of retaining the repressive histone modification state through multiple DNA replications, independent of the DNA sequence and recruitment factors. Indeed, from as early as 1996, the inheritance of stable epigenetic states (though both mitosis and meiosis) was observed at the mating type loci of *S. pombe*, and a then unknown self-templating nucleoprotein complex was theorised to mediate this inheritance [63]. Later studies identified histones as the nucleoprotein complex, and CLRC silencing as the templating pathway, and recently the DNA sequence-independent inheritance of the heterochromatic state was definitively shown by a pair of breakthrough studies in *S. pombe* [58,59]. Both studies used an inducible system to establish heterochromatin at a genomic locus of choice whereby the Clr4p enzyme was tethered to an exogenous tetracycline repressor (TetRp) protein. By integrating TetRp-binding sites into the *S. pombe* genome, the TetRp–Clr4p initiator could be targeted to a locus (in the absence of tetracycline), where it would establish an ectopic heterochromatic domain in tandem with the cell’s endogenous machinery. The addition of tetracycline then released TetRp-Clr4p from the binding site and allowed the authors to determine whether the silent chromatin state could be inherited without the initiator, at naturally euchromatic loci, monitored by the silencing of the *ade6^+^* reporter gene. One group found that *ade6+* remained in an OFF state for 10 generations, which could be increased to at least 50 generations in the background of an *epe1Δ* deletion [59]. Epe1p is a JmjC histone demethylase that targets Histone H3K9me, thus antagonising heterochromatisation in *S. pombe* [64]. The second group reported significant epigenetic inheritance of this ectopic locus and reported a loss of *ade6*+ silencing of only 4% cell division, again in an *epe1Δ* deletion strain [58]. The targeting of this system to euchromatic loci is a key aspect of both studies, showing that although the DNA sequence primarily determines the location of silent chromatin, the maintenance and inheritance of heterochromatin can occur regardless of the underlying sequence. The fact that the CLRC complex is instrumental in H3K9me-dependent heterochromatin formation in *S. pombe* enabled the development of this inducible system, although a similar approach could potentially be used to investigate SIR-mediated silencing in *S. cerevisiae*.

## The preservation of histone modifications during S-phase

The heritability of a chromatin mark is often considered to be the defining factor that identifies it as epigenetic, and a discussion of epigenetics is incomplete without consideration of how histone modifications are/can be inherited. For a histone modification to be maintained through DNA replication, parental histone proteins must be transferred to nascent DNA, without the loss of the mark. Given that S-phase demands a two-fold increase in nucleosome number, parental histones can constitute at most half of the post-replication nucleosomes (reviewed in [65]). Early pulse-chase experiments revealed that the majority of parental histones do indeed remain bound to nascent DNA [66]. This observation has led to a model whereby parental histones serve as a template for the transmission of epigenetic information to new histones, which would be necessary to prevent the dilution of their modifications, and the loss of parental chromatin states. Accordingly, the copying of a histone mark from parental to new nucleosomes is carried out through a read-write mechanism whereby the co-operation between a ‘reader’ protein, which recognises and binds histone modifications, and a ‘writer’ protein, propagates the modification to neighbouring histone proteins (reviewed in [67–70]), and [56].

Histones H3 and H4 have been identified as the most likely carriers of epigenetic information, based on their aforementioned modifications and low replication-independent turnover rates compared with those of histones H2A and H2B [71]. The inheritance of histone H3 and H4 during S-phase has been assessed in *S. cerevisiae* using epitope-tagged histone H3 that distinguishes parental from newly synthesised H3 [72]. This experiment confirmed earlier studies suggesting that parental histone H3 and H4 are randomly distributed to both daughter DNA strands during replication ([66,73] and reviewed in [74]). Furthermore, this study reported that parental histone H3 is retained in budding yeast within ∼400 bp of its original position during DNA replication over each cell division [72] thus providing a mechanism that would facilitate the ‘read-write’ model of modification inheritance at a gene-specific level. In 2016 it was shown that the histone marks, H3K4 methylation and H4K16 acetylation are very quickly re-established on nascent DNA strands following the passage of the replication fork, suggesting that these marks in particular might contribute to epigenetic inheritance in yeast [75].

## Nucleosome dynamics during DNA replication

**Figure 3:**
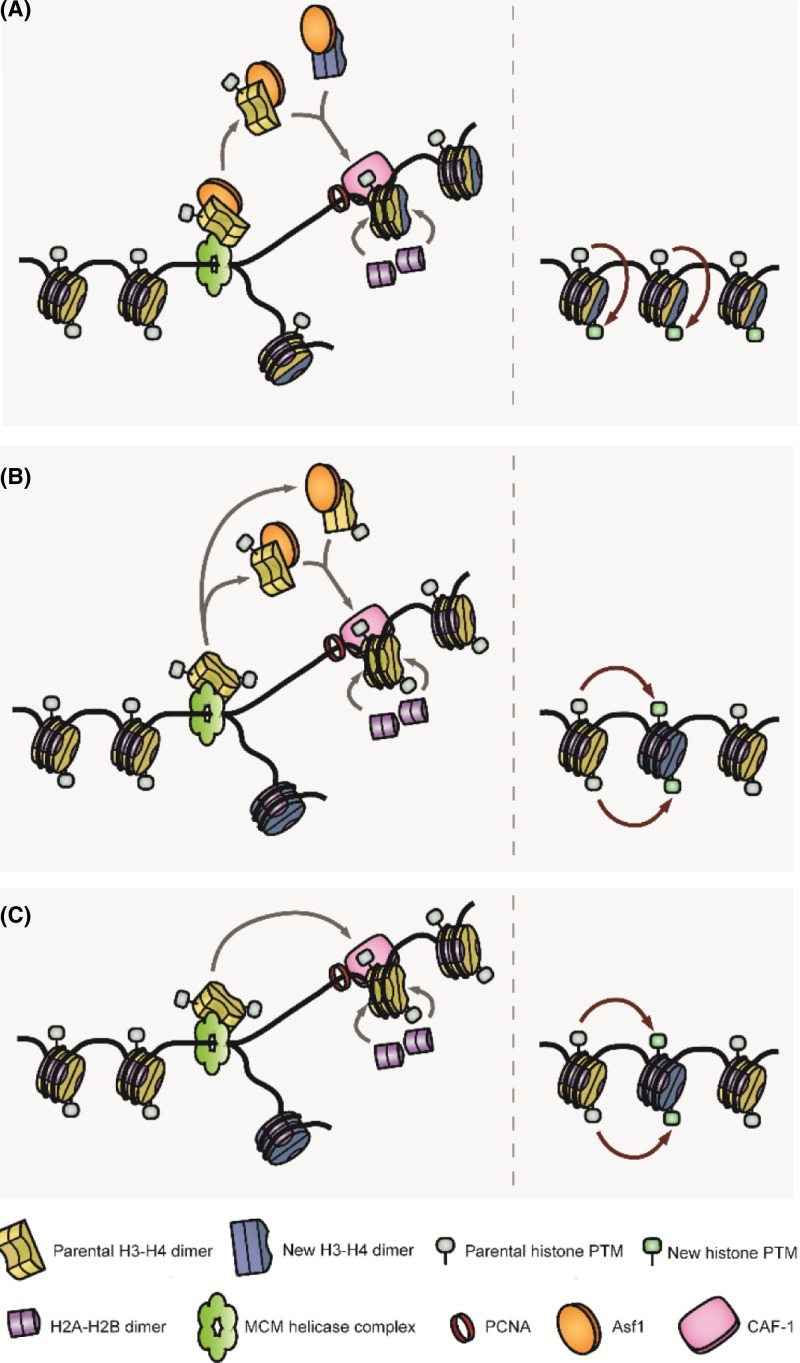
The inheritance of histone modifications (**A**) Parental (H3–H4)4 tetramers are split and reassembled into tetramers consisting of both old and new histone proteins on nascent DNA, this semiconservative inheritance would necessitate the intranucleosomal copy of histone PTMs. (**B**) Parental (H3–H4)_2_ tetramers are transiently disrupted before being reassembled on daughter DNA strands, also requiring internucleosomal templating. (**C**) Intact (H3–H4)_2_ tetramers are directly transferred on to nascent DNA, requiring the internucleosomal templating of histone PTMs. Note that these models are not mutually exclusive, numerous replisome components and chaperone proteins have been shown to be capable of binding both H3–H4 dimers and (H3–H4)_2_ tetramers (see text). Abbreviation: PTM, post-translational modification.

The molecular details of how parental histones are transferred on to nascent DNA during DNA replication however remain unclear. It is known that the assembly of newly synthesised histones on DNA involves the deposition of an (H3–H4)_2_ tetramer, followed by a pair of H2A–H2B dimers [76], and therefore it is unlikely that parental nucleosomes are inherited in an octameric form (reviewed in [77]). However, two models exist to describe the inheritance of parental histone H3 and H4. In the semiconservative model of nucleosome assembly, parental (H3–H4)_2_ tetramers are split into two H3–H4 dimers, which are partnered by a newly synthesised H3–H4 dimer on daughter strands [78] (Figure 3A). Under this model the parental H3–H4 dimer could then act as a template for the ‘intranucleosome’ restoration of histone modifications. In support of this it was found that the histone chaperone Asf1p, which localises to the DNA replication fork, binds an H3–H4 dimer with high affinity and blocks the formation of the (H3–H4)_2_ tetramer [79]. In *S. cerevisiae*, Asf1p passes H3–H4 dimers to the chaperone CAF-1, which assembles (H3–H4)_2_, and deposits the tetramer on to nascent DNA [80]. Asf1p also plays a role in the Rtt106p-mediated deposition of (H3–H4)_2_ tetramers [81]. However, this activity may be exclusive to newly synthesised histones given that (i) histone-tagging studies on *S. cerevisiae* found that tetramer splitting rarely occurs during DNA replication [82] and, (ii) residues on (H4–H3)_2_ tetramers can be asymmetrically modified in eukaryotic cells, which undermines the idea of ‘intranucleosome’ templating [83]. If Asf1p does interact with parental histones, it may serve to disassemble and faithfully reassemble the histones during the passage of the replication fork (Figure 3B).

The majority of evidence to date supports a conservative model of nucleosome assembly (reviewed by [77]), whereby the (H3–H4)_2_ tetramer is not split, necessitating instead the ‘internucleosome’ copying of histone modifications (Figure 3C). Such a mechanism could function well for the maintenance of homologous stretches of nucleosome modifications, however heterologous and/or niche histone modifications would prove difficult to conserve, unless there was an interface between the nucleosomes on separate daughter DNA strands. The stochastic nature of parental histone distribution also means that stretches of asymmetric distribution will arise, resulting in regions of nascent DNA containing exclusively new or old histones, which may limit the fidelity of histone modification maintenance. Given that repressive histone modifications do not directly silence genes (rather, the resultant chromatin structure inhibits transcription), Xu *et al.* [84] proposed the ‘buffer model’ of epigenetic inheritance, in which gene silencing is caused by a threshold quantity of repressive histone modifications over a broad genomic region, rather than the precise action of any single histone mark. This model of epigenetic repression could tolerate aberrations that may occur during DNA replication, and would be likely to provide a more robust epigenetic state over a long timescale. A combination of the buffer model and the conservative model of nucleosome assembly form a theoretical mechanism for the long-term maintenance of epigenetic marks in yeast.

A recent advancement in our understanding of the propagation of epigenetic states through S-phase in *S. pombe*, has identified the chromatin remodeler, Fft3p, as a key regulator in maintaining a heterochromatic state at the mating type (mat) locus and telomeres [85]. In this study Grewal and colleagues have found that Fft3p suppresses nucleosome turnover at these loci thus serving to preserve the heterochromatin-associated H3K9 methylation modification [85]. Furthermore, in *fft3Δ* mutant cells, there is cumulative loss of heterochromatin silencing with each round of cell division, indicating a role for Fft3p in maintaining heterochromatin specifically in cycling cells. Indeed, the authors detected an interaction with Fft3p and replisome components, placing it at the replication fork. However, it is unclear how Fft3p co-ordinates with histone chaperones and histone modification machinery to suppress the loss of parental histones and promote the faithful inheritance of H3K9 methylation. Fft3p is a member of a highly conserved group of ATP-dependent chromatin remodelers [86] so it will be interesting to uncover whether histone turnover repression is an evolutionarily conserved function for other Fftp3 homologues.

## The future of yeast epigenetics: filling the gaps and beyond

Although the epigenetic mechanisms of yeast are perhaps the best understood among eukaryotic systems, we have alluded to the many gaps that still remain in our knowledge. We would also argue that a systems biology approach is needed to understand epigenetics at a more global level, so that we can begin to tease apart how cross-talk between different histone modification marks (see [69]), and/or between the same marks in different regions of the genome, dictate chromatin function. Towards this end, chromosome conformation capture techniques (3C) and Hi-C technology is providing us with spatial information of genome organisation that can be incorporated into models of how chromatin marks are restricted spatially. Additionally, while the mitotic inheritance of epigenetic states has been addressed to some extent, the same cannot be said for the inheritance of histone PTMs through meiosis and sexual reproduction in yeast. Limited studies have identified a role for the histone PTMs H3K56 acetylation and Histone H2A Serine121 phosphorylation during budding yeast meiotic division [87,88], however a lot remains to be explored, not least whether these modifications influence the faithful transmission of heterochromatic silent states through meiosis. And lastly, rDNA silencing in yeast is molecularly distinct from telomeric silencing and the molecular mechanisms that dictate the maintenance of this heterochromatic locus for the most part, remain elusive.

Because of their use as a model system, understandably studies on *S. cerevisiae* and *S. pombe* dominate the field of yeast epigenetics. However, pathogenic yeast have evolved unique epigenetic programmes central to their biology that warrant investigation. These epigenetic programmes provide fungal pathogens with phenotypic plasticity that allows them to respond to environmental cues and survive in a human host. For example, the pathogenic species *Candida albicans* can switch between two distinct epigenetic states (termed as white and opaque), which vary in morphology, metabolism and most importantly, virulence. These states may well contribute to the yeast’s ability to invade the human body [89]. The relationship between epigenetic inheritance and fungal disease is an underexplored topic in yeast epigenetics that merits attention, especially given our current limited arsenal of antifungal therapies to combat against these pathogens.

## Abbreviations

ARC: argonaute siRNA chaperone complex
BAH: bromo-adjacent homology domain (Sir3p)
CLRC: Clr4 methyltransferase complex
H3K79me: histone H3 lysine 79 methylation
H3K9me: histone H3 lysine 9 methylation
H4K16ac: histone H4 lysine 16 acetylation
its: increase in telomeric silencing
lts: loss of telomeric silencing
OAADPr: O-acetyl-ADP-ribose
PTM: post-translational modification
RDRC: RNA-dependent RNA polymerase complex
RNAi: RNA interference
RNAPII: RNA Polymerase II
SIR: silent information regulator
TetRp: tetracycline repressor
TPE: telomeric position effect
